# Comparative efficacy of aloe vera mouthwash and chlorhexidine on periodontal health: A randomized controlled trial

**DOI:** 10.4317/jced.53033

**Published:** 2016-10-01

**Authors:** Swathi Vangipuram, Abhishek Jha, Mamtha Bhashyam

**Affiliations:** 1 Senior lecturer,Department of Public health dentistry, krishnadevaraya college of Dental sciences and Hospital, Bangalore, Karnataka; 2 Senior Lecturer, Department of Public Health Dentistry, New Horizon Dental College, Bilaspur, Chhattisgarh; 3 Post Graduate Student, Department of Public health dentistry, V S dental College and Hospital Bangalore, Karnataka, India

## Abstract

**Background:**

With introduction of many herbal medicines, dentistry has recently evidenced shift of approach for treating many inflammatory oral diseases by using such modalities. Aloe vera is one such product exhibiting multiple benefits and has gained considerable importance in clinical research recently.

**Aim:**

To compare the efficacy of Aloevera and Chlorhexidine mouthwash on Periodontal Health.

**Material and Methods:**

Thirty days randomized controlled trial was conducted among 390 dental students. The students were randomized into two intervention groups namely Aloe Vera (AV) chlorhexidine group (CHX) and one control (placebo) group. Plaque index and gingival index was recorded for each participant at baseline, 15 days and 30 days. The findings were than statistically analyzed, ANOVA and Post Hoc test were used.

**Results:**

There was significant reduction (*p*<0.05) in the mean scores of all the parameters with Aloe Vera (AV) and chlorhexidine group. Post hoc test showed significant difference (*p*<0.000) in mean plaque and gingival index scores of aloe Vera and placebo and chlorhexidine and placebo group. No significant difference (*p*<0.05) was observed between AloeVera and chlorhexidine group.

**Conclusions:**

Being an herbal product AloeVera has shown equal effectiveness as Chlorhexidine. Hence can be used as an alternative product for curing and preventing gingivitis.

** Key words:**Aloe vera, chlorhexidine, dental plaque, gingivitis.

## Introduction

Recent advances in the field of dentistry have seen a trend of the use of various herbal and natural products for the treatment of various oral diseases and conditions. Many plant products have been used with great effectiveness for cleaning teeth and as an-ti-microbial agents. Such products can offer a suitable alternative to antibiotics and antimicrobials used for same purpose (1). Another advantage of using herbal medicine is long term usage of such products possesses lesser chance of side effects (2). Aloe Vera is one such product exhibiting multiple benefits and has gained considerable importance in clinical research (3). Aloe Vera is a succulent, cactus like plant belonging to the Aloe cease family (subfamily of the Asphoelaceae). Among more than 400 aloe species, Aloe barbadensis Miller and Aloe aborescens are the most accepted species for various medical, cosmetic, and pharmaceutical purposes (4,5). The composition of Aloe Vera is very complex, consisting of 75 different ingredients including minerals, enzymes, sugars, anthraquinones, lignin, saponins, sterols, amino acids and salicylic acid (6). Gjerstad *et al.* found that the leaves of aloe vera plant contained 99.5% water and 0.0013% protein (7). The pharmacokinetics actions of Aloe Vera gel as studied in *in vitro* and *in vivo* include anti-inflammatory, antibacterial, antioxidant, immune-boosting and hypoglycemic properties (8,9). Aloevera has shown its anti-microbial potential against *Streptoccocus pyogenes* and *Streptococcus faecalis* (10). Three aloesin derivatives from aloe (namely isorabaichromone, feruoylaloesin, and p-coumaroylaloesin) showed potent free radical and superoxide anion-scavenging activities (11,12). Geetha *et al.* in 2012 conducted a study on Aloe Vera and highlights its property when used as a medicament in the periodontal pocket (13). George D, Bhat SS, conducted a study to compare the antimicrobial efficacy of aloe Vera tooth gel and two popular tooth paste and concluded that aloe Vera tooth gel was as effective as two commercially popular tooth pastes in controlling all the organisms (14).

Periodontal diseases are chronic infectious diseases characterized by a bacterial challenge that can provoke a destructive host response, leading to clinical attachment loss destruction of tooth supporting structures and ultimately lead to possible tooth loss (15-17). It is well established that supragingival plaque is the cause of gingivitis and plays a primary role in the initiation of pe-riodontitis, which further leads to progression into chronic periodontitis, formerly known as “adult periodontitis” or “chronic adult periodontitis”, is the most prevalent form of periodontitis (18). Culture of plaque microorganisms from sites of chronic periodontitis reveals high percentages of anaerobic (90%) and gram negative (75%) bacterial species (19). At present mechanical methods of dental plaque removal are widely regarded as being a highly effective means of helping to control progression of dental caries and periodontal diseases (20). Mouth rinses are generally considered as adjuncts to oral hygiene and widely used in the delivery of therapeutic agents to the teeth and gums. Chlorhexidine is regarded as benchmark control in plaque removal, but it has numerous side effects on chronic usage such as staining of the teeth and the tongue, altered taste sensation, and increased calculus formation often deters its usage (20). Hence, there is a need to develop a naturally occurring, indigenous and cost-effective oral hygiene aid. One such aid could be in the form of Aloe Vera extract. Although the medicinal use of Aloe Vera has been reported, not much literature is available regarding its use in the field of dentistry as mouth rinse.

Hence the purpose of this study was to evaluate efficacy of Aloe Vera mouthwash on the dental plaque and gingivitis and comparing it with the bench mark control chlorhexidine and placebo.

## Material and Methods

The present randomized controlled trial was conducted for 30 days to compare the efficacy of Aloe Vera and Chlorhexidine in preventing plaque accumulation and gingival inflammation.

-Study population and sample size

**Figure 1:**
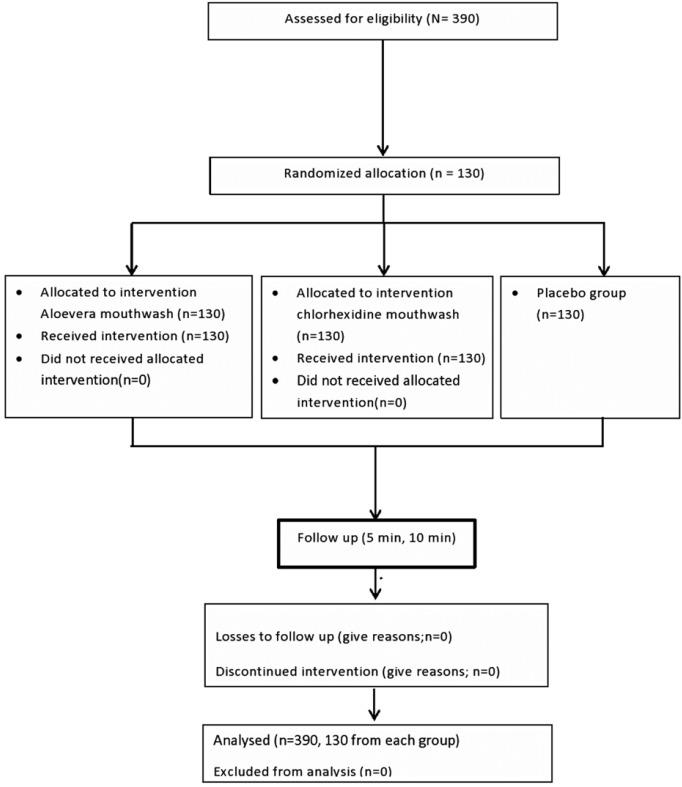
Showing allocation of study participants according to CONSORT 2010.

Three ninety (390) under and postgraduate dental students among the age group of 18-40 years studying in New Horizon Dental College and research institute formed the study population (Fig. 1). The study was conducted in department of public health dentistry. A pilot study was done among 15 subjects, for 15 days in each group to know the constraints, feasibility and effect size. Sample size was calculated by keeping Power of study (1-β) = 80, Effect size 0.353, Confidence interval 0.05 sample came out to be 126 each group and was rounded to 130 in each group.

The ethical approval was obtained from ethical review board New Horizon Dental College and research institute NHDCRI, Bilaspur, and written informed consent was taken from each participants. Study was conducted in accordance with the declaration of Helsinki for Biomedical research involving human subject.

Inclusion criteria was study subjects with Plaque Index score more than 1.9, Gingival Index score more than 1.1, with no ongoing dental treatment, antibiotic or anti-inflammatory drug therapy for the past 3 months. Exclusion criteria was subjects with any of history systemic diseases/conditions, fibrotic gingival enlargement and smoking were excluded from the study. Those subjects who had used antibiotics or mouthwash for 5 consecutive days or corticosteroids in the past 30 days and who had a history of sensitivity to any mouthwash or used removable prostheses or an orthodontic appliance were excluded from the study

-Methodology 

The Study was conducted for 30 days. The study participants were randomly allocated into the three study groups (130 in each groups) through lottery method. Individuals were identified by code numbers throughout the study. The Three groups formed were i) Aloe Vera mouth wash group ii) Cholrhexidine (0.12%) mouth wash group iii) Placebo group. All three types of mouthwashes were kept in similar container after preparation and same flavor of spearmint was added to each solution to blind the subjects in which group they fall (single blinding). All mouth rinse bottle were coded and was handed over to study participants by an investigator-1 not participating in the clinical examination and data analysis. (Double blinding). Investigator-2 too was unaware of the mouthwash allotted, who recorded plaque and gingival index scores. (triple Blinding). All clinical parameters were recorded at base line, 15 days and 30 days. For oral hygiene all participants were given similar set of toothbrush (Thermoseal Ultra-soft ICPA Health Products Limited) and toothpaste ( Sensodent K Toothpaste, Warren Pharmaceuticals, containing potassium nitrate as key ingredient, without any antiplaque agent), each participants were asked to brush 2 minutes in morning, to nullify any confounding effect of oral hygiene measures.

1) Group 1 (n=130) - was given Aloe Vera mouthwash and instructed to use 10 ml twice a day for 30 days.

2) Group 2 (n=130) was given Chlorhexidine and instructed to use 10 ml twice a day for 30 days

3) Group 3 (n=130) was the placebo group and distilled water was given as the mouthwash. They were also instructed to use 10 ml of distilled water twice a day as mouthwash for 30 days.

-Preparation of Aloe vera mouthwash 

Aloe Vera juice was provided to study participants. Aloe vera juice consisted of 99% aloe juice, 0.2% preservative , 0.001% Spearmint flavor, and sweetened with sorbitol. The placebo solution and the control were taste-matched, with identical astringency and consistency.

-Study tool

It consisted of a proforma divided into two parts. Part one consisted of structured interview which recorded demographic data, oral hygiene practices and past medical and dental history of the participants. The second part consisted of clinical assessment of gingival health by using gingival index given by Loe H and Silness J in 1963 and plaque index by Silness P and Loe H in 1964 at baseline, 15 and 30 days which formed the dependent variables. Gingival index was recorded on Index teeth using mouth mirror and probe based on degree of inflammation score 0,1,2,3 was allotted to all index teeth and subsequently final score was allotted for individual participant. Similarly plaque index was recorded for each index teeth and score 0,1,2,3 was given to each index tooth and final score was calculated for each participant. Two examiners were trained and calibrated to record plaque and gingival index. The examiners were blinded to group allocation. The inter and intra examiner reliability was 0.90 and 0.86 respectively.

-Statistical analysis

The data were analyzed using SPSS version 17. ANOVA followed by Tukey post-hoc were used for analysis. *p*-Value of 0.05 was taken to be significant.

## Results

A total of 390 students participated in the present study out of which 210 were females and 180 were male. Mean age of study participants was 23 years. 240 participants were under graduate students, 98 were in Internship and 52 were postgraduate students, all participants reported using toothbrush and tooth paste as the principal method of oral hygiene maintenance, while only 105 reported using any other oral hygiene aid such as dental floss or mouth wash in past ( Table 1). In follow up there was no loss of sample in follow-up, because all of the study participants were students of same institution and the nature of study was explained to them at the start of study. Mean plaque and gingival index were recorded for all three groups and reduction of plaque and gingival scores was seen in all groups ( Table 2). ANOVA was carried out to assess the intra- and inter-group variations for plaque and gingivitis respectively. At base line there was no significant difference in the clinical parameters but the difference were significant at the 15 day and 30 day follow up between and within group for both plaque and gingival index in both Chlorhexidine and Aloe Vera group (*p*-value <0.05). When compared to 15 day follow-up, 30 day follow up showed highly significant difference for both the index in both groups ( Table 3). Multiple comparisons were obtained by Post-hoc LSD. There was a progressive reduction in both plaque and gingival index values at subsequent follow ups but difference in the decrease in scores between aloe vera and chlorhexidine group was not statistically significant as shown in by post-hoc test, hence showing both Chlorhexidine and Aloe Vera equally efficient in treating gingivitis. However, the difference between Aloe vera and the placebo group and chlorhexidine and the placebo group was statistically significant (*P* <.05) ( Table 4).

**Table 1:**
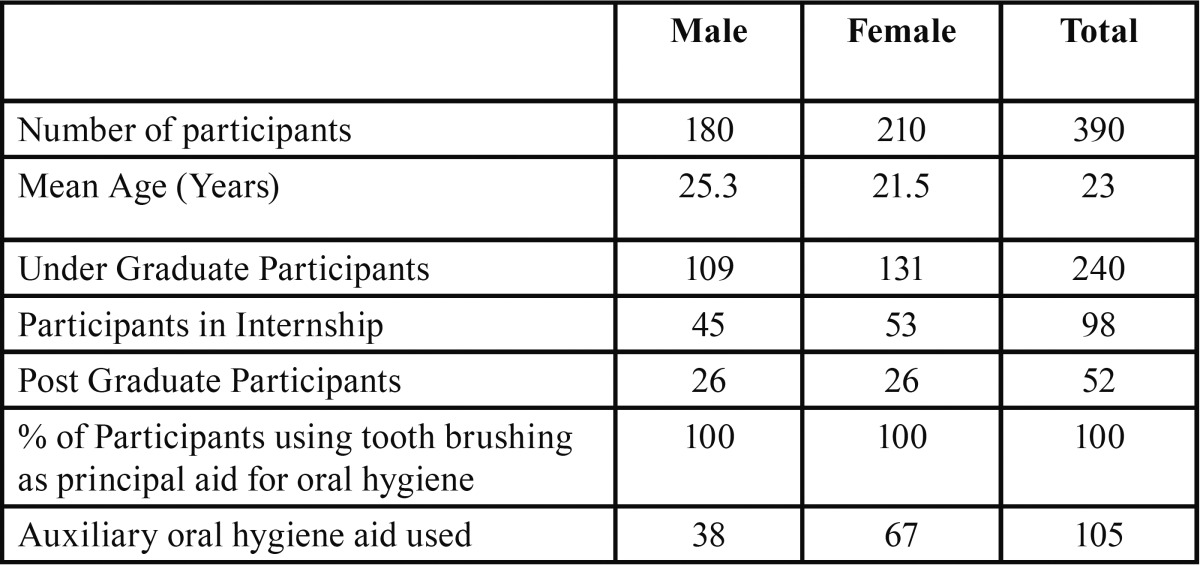
Distribution of study participants.

**Table 2:**
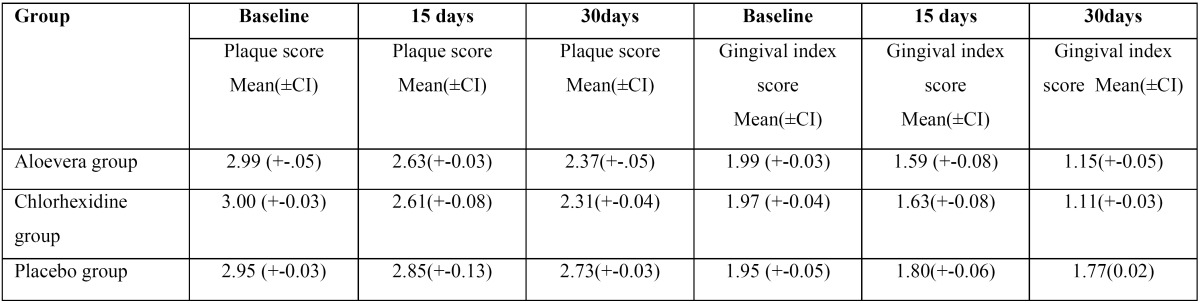
Mean Values of plaque and Gingival Index of three groups (AV, CHX and placebo) at different time intervals.

**Table 3:**
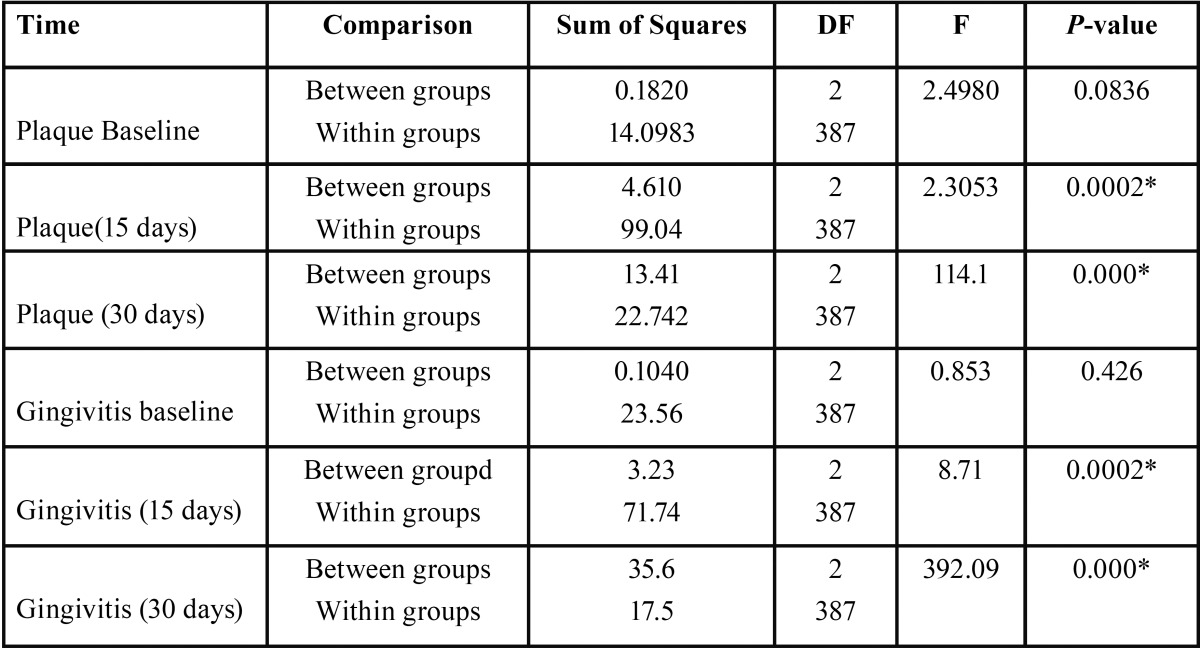
ANOVA of three study groups for intra- and inter-group variations for plaque and gingivitis; (ANOVA, *p*-Value< 0.05*).

**Table 4:**
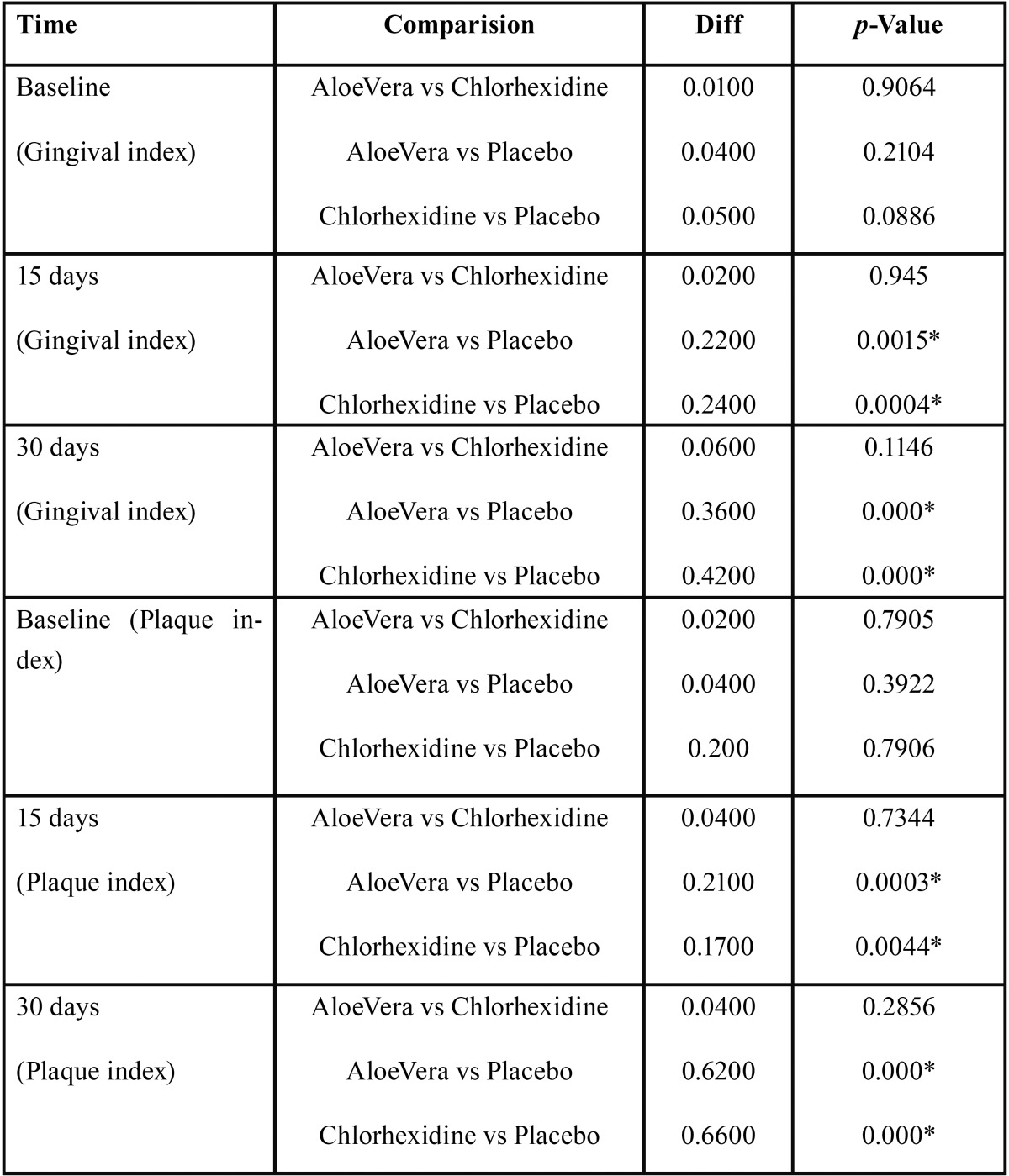
Post-hoc test for Multiple comparision for Gingival and plaque index scores for all groups. (Tukey Post-Hoc Test. *p*-Value<0.05*).

## Discussion

The present randomized controlled trial was conducted to assess efficacy of Aloe Vera mouthwash in preventing plaque accumulation and gingival inflammation. Aloe Vera is a potential anti-bacterial agent which is said to be very effective in fighting the bacteria and preventing gingival and periodontal disease (21). It reduces edema of the soft tissues and consequently reduces the bleeding of the gums. It exhibits strong antiseptic action in gingival pockets where normal cleaning is difficult (22,23). Chlorhexidine, sodium hypochlorite, amine fluoride and cetylpyridinium chloride are widely used as mouthwashes and irrigating agents that can inhibit the growth of potentially pathogenic oral bacteria (24). Although these antimicrobial agents are widely used, side effects such as immediate hypersensitivity reactions, toxicity, tooth staining and other side effects have been reported. Moreover, it has been reported that chlorhexidine and sodium hypochlorite possess cytotoxicity toward human periodontal ligament cells, inhibit protein synthesis, and affect mitochondrial activity, thus having detrimental effects on oral tissues (25). The natural phytochemicals isolated from medicinal plants used in traditional medicine have been considered useful alternatives to traditional allopathic drugs. Many medicinal plants and their products are widely used for prevention and treatment of oral conditions, and among them Aloe Vera is of particular interest and has been used therapeutically (11-13).

The low plaque index observed in study subjects could be explained by the fact that Aloe Vera is a good antibacterial. Heggers *et al.* showed its antimicrobial properties against *Candida albicans*, *Streptococcus pyogens*, *Streptococcus fecalis* (26). Noskova used Aloe Vera to treat early stages of periodontitis and got good results (27). Both chlorhexidine and aloe Vera mouthwashes had reduced plaque scores significantly. The results of present study are in accordance with Karim *et al.* who found significant reduction in plaque scores after using Aloe Vera mouthwash for 30 and 22 days (28).

The reduction in gingival index scores can be attributed to components of Aloe Vera. Aloe Vera extracts have shown inhibition of the cyclooxygenase pathway and reduces prostaglandin synthesis form arachidonic acid, thus reducing inflammation. Vitamin C present in Aloe vera is involved in collagen synthesis, increases concentration of oxygen at the wound site because of dilation of blood vessels (29). The results of this study were in agreement with those presented by Bhat *et al.* who used sub gingival delivery of Aloe Vera gel in chronic periodontitis treatment. There was a significant reduction in gingival index, plaque index (13).

Furthermore, the results of present study are in agreement with those of Villalobes *et al.* who observed a significant reduction in plaque and gingivitis after a 30 day use of mouth rinses containing Aloe Vera associated with tooth brushing (30). Also, de Olivera *et al.* found that both dentifrices containing Aloe Vera and dentifrice containing fluoride resulted in significant reduction of plaque and gingivitis, but no statistical significant difference was observed between them (31). The results were also consistent with Pradeep *et al.* who found toothpaste containing Aloe Vera showed significant improvement in gingival and plaque index compared with placebo dentifrice (32).

## Conclusions

It can be concluded from the present study that Aloe Vera mouthwash is equally effective as chlorhexidine in reducing plaque and gingivitis. It promises to be a better preventive home care therapy in developing countries like India where accessibility, affordability, availability and sustainability are important issues. Further studies should be carried out with larger samples, varying time period of trial to establish its efficacy in prevention of periodontal problems and open new doors in the field of research in oral health care.
